# Serum Autoantibodies in Chronic Prostate Inflammation in Prostate Cancer Patients

**DOI:** 10.1371/journal.pone.0147739

**Published:** 2016-02-10

**Authors:** Bettina Schlick, Petra Massoner, Angelika Lueking, Pornpimol Charoentong, Mirjam Blattner, Georg Schaefer, Klaus Marquart, Carmen Theek, Peter Amersdorfer, Dirk Zielinski, Matthias Kirchner, Zlatko Trajanoski, Mark A. Rubin, Stefan Müllner, Peter Schulz-Knappe, Helmut Klocker

**Affiliations:** 1 Division of Experimental Urology, Dept. of Urology, Medical University of Innsbruck, Innsbruck, Austria; 2 ONCOTYROL, Center for Personalized Cancer Medicine, Innsbruck, Austria; 3 Protagen AG, Dortmund, Germany; 4 Department of Pathology, Medical University of Innsbruck, Innsbruck, Austria; 5 Division of Bioinformatics, Medical University of Innsbruck, Innsbruck, Austria; 6 Department of Pathology and Laboratory Medicine, Institute of Precision Medicine, Weill Medical College of Cornell University, New York, NY, United States of America; 7 TARGOS Molecular Pathology GmbH, Kassel, Germany; King's College London, UNITED KINGDOM

## Abstract

**Background:**

Chronic inflammation is frequently observed on histological analysis of malignant and non-malignant prostate specimens. It is a suspected supporting factor for prostate diseases and their progression and a main cause of false positive PSA tests in cancer screening. We hypothesized that inflammation induces autoantibodies, which may be useful biomarkers. We aimed to identify and validate prostate inflammation associated serum autoantibodies in prostate cancer patients and evaluate the expression of corresponding autoantigens.

**Methods:**

Radical prostatectomy specimens of prostate cancer patients (N = 70) were classified into high and low inflammation groups according to the amount of tissue infiltrating lymphocytes. The corresponding pre-surgery blood serum samples were scrutinized for autoantibodies using a low-density protein array. Selected autoantigens were identified in prostate tissue and their expression pattern analyzed by immunohistochemistry and qPCR. The identified autoantibody profile was cross-checked in an independent sample set (N = 63) using the Luminex-bead protein array technology.

**Results:**

Protein array screening identified 165 autoantibodies differentially abundant in the serum of high compared to low inflammation patients. The expression pattern of three corresponding antigens were established in benign and cancer tissue by immunohistochemistry and qPCR: SPAST (Spastin), STX18 (Syntaxin 18) and SPOP (speckle-type POZ protein). Of these, SPAST was significantly increased in prostate tissue with high inflammation. All three autoantigens were differentially expressed in primary and/or castration resistant prostate tumors when analyzed in an inflammation-independent tissue microarray. Cross-validation of the inflammation autoantibody profile on an independent sample set using a Luminex-bead protein array, retrieved 51 of the significantly discriminating autoantibodies. Three autoantibodies were significantly upregulated in both screens, MUT, RAB11B and CSRP2 (p>0.05), two, SPOP and ZNF671, close to statistical significance (p = 0.051 and 0.076).

**Conclusions:**

We provide evidence of an inflammation-specific autoantibody profile and confirm the expression of corresponding autoantigens in prostate tissue. This supports evaluation of autoantibodies as non-invasive markers for prostate inflammation.

## Introduction

The prostate gland is a site of frequent benign and malignant disease with age as the main risk factor [1, 2]. As a downside of the constantly increasing life expectancy the incidence of prostate diseases, such as prostate cancer, prostatic hyperplasia (BPH) and prostatitis is rising as well and these diseases represent a growing medical and social problem but also an increasing economic burden [3–6]. Frequently, histopathological analysis of prostate biopsies and surgical specimens reveals inflammation associated with prostate disease, in most cases an asymptomatic, “chronic” inflammation characterized by histological alterations and immune cell infiltrates [7–10]. Chronic inflammation may be one of the drivers of prostate disease progression and a major contributing factor to false-positive prostate specific antigen (PSA) prostate cancer testing [11–15].

The source of intraprostatic inflammation is yet not fully uncovered, infection, autoimmunity, cell injury, hormonal variations, or dietary factors might contribute. As much as the cause for prostatitis is remaining a challenge for further investigations, its relevance to pathological processes is unclear [16]. Studies suggest a contribution of chronic inflammation to carcinogenesis and development of prostate disease [17, 18]. Particularly, proliferative inflammatory atrophy (PIA) that is considered a prostate cancer precursor lesion, is associated with inflammatory immune cell infiltrates, which stimulate proliferation, support carcinogenesis via enhanced oxidative stress and cellular damage and pioneer malignant degeneration [19]. Although most studies addressed the impact of inflammation on carcinogenesis and tumor progression, this phenomenon is not restricted to cancer, immune cell infiltrates are also found frequently in hyperplastic or even in histologically normal prostate tissue [17].

In light of the need to continue efforts clarifying the impact of inflammation on prostatic disease, easily accessible biomarkers for prostate inflammation would be a great help. Moreover, such markers that allow to a less invasive way for the diagnosis, prognosis and treatment monitoring of chronic prostatitis is required for improving patient care, increase the specificity of the PSA test for prostate cancer detection and lead to an improvement of prostate cancer management. In this context autoantibodies are important types of marker molecules, as they are linked to the activity of the immune system. Antibodies have ideal properties as potential markers, they are very stable and do not undergo short-term variations in concentration, they are easily accessible via serum or plasma samples and in every clinical laboratory there are highly sensitive detection methods available for their quantification. For cancer patients it was convincingly demonstrated that an anti-tumor immune response can be triggered [20, 21]. Autoantibodies against cancer antigens have been identified in patients with different solid tumor entities such as for instance tumors of the breast [22, 23], head and neck [24, 25], lung [26, 27], esophagus [28], colon [29] and prostate [26, 30, 31].

Recently, we employed protein microarrays for autoantibody profiling in the blood of prostate cancer patients and non-cancer controls. We identified a panel of prostate cancer associated autoantibodies [31]. Extending these studies we here present the identification and validation of autoantibodies associated with prostate immune cell infiltrates in prostate cancer patients. In addition, we evaluated the expression pattern of corresponding autoantigens in malignant and non-malignant prostate tissue and enquired whether mutations might trigger autoantibodies.

## Material and Methods

### Retrospective sample cohort

Serum samples were obtained from the Prostate Cancer Bioresource of the Department of Urology, Innsbruck Medical University. The blood samples had been collected within the framework of the Tyrolean prostate cancer early detection program and were stored at -80°C until use [32]. Informed consent was obtained from all patients and the study was approved by the Ethics Committee of the Medical University of Innsbruck (Study AM 3174, amendment 2). All samples were obtained prior to radical prostatectomy surgery from patients with biopsy-proven, clinically localized prostate cancer, who were at least 40 years old and who had received no previous prostate-cancer therapy. Patient selection for low and high inflammation cases was based on analysis of whole prostate gland specimens for infiltrating lymphocytes. A cohort of 70 patients (38 high, 32 low infiltration) for the screening study and 63 patients (33 high, 30 low infiltration) for the cross-validation study were recruited.

### Autoantibody profiling

The assays used for autoantigen profiling, a low-density protein array and a Luminex-bead protein array, respectively, were established as broadly applicable techniques and not specifically for prostate cancer only. Whereas the protein array technique was applied for the initial screening study, the later established Luminex-bead technique was used for an independent cross-validation study.

Selection of included autoantigen proteins was based on our previous autoantibody screens in prostate cancer [31] and autoimmune diseases [33–35], and on published autoantigens found associated with cancer [36–42]. In addition, protein production efficacy, protein quality, and assay performance criteria were considered for the final selection of autoantigen proteins. 4012 proteins were selected for the protein array, 3061 proteins for the Luminex-bead protein array. Autoantigen panels are listed in Supporting Information **(S1 Table)**. The bead protein assay was grouped into 8 subarrays as the maximal available number of individually color-coded LuminexMagPlex^TM^ beads (Luminex, MV‘s-Hertogenbosch, The Netherlands) is 500. The Luminex-bead autoantibody assay was established for a broad application, independent of the initial prostate low/high inflammation screening results and therefore does not exactly match the initial autoantigen panel. Based on the corresponding gene ID’s, 84% of the autoantigen proteins of the Luminex-bead assay were also represented in the protein microarray.

Autoantigen proteins were expressed in *E*. *coli* and recombinant proteins were purified using a His-tag affinity purification procedure. Generation of planar protein microarrays was carried out as described previously [31]. Briefly, proteins were spotted in quadruplicates on nitrocellulose-coated FAST slides (GE Healthcare). Mouse and human IgGs at different concentrations were added to be used as immune detection controls and for data normalization. An automated station (HS 4800 Pro, Tecan) was used to perform the microarray autoantibody analysis. Array slides were blocked with 2% (w/v) bovine serum (BSA) in TBS containing 0.1% (v/v) Tween 20 (TBST) and blood serum samples were added in a 1:100 dilution in 2% (w/v) BSA/TBST. After incubation at room temperature for 16 hrs secondary (mouse-anti-human-IgG, 1:5000, Sigma) and tertiary (Cy3-labeled donkey anti-mouse IgG, 1:500, Jackson ImmunoResearch) antibody incubation steps were carried out in 2% (w/v) BSA/TBST at room temperature for 1h. After each incubation step, slides were washed 3 times with TBST. Processed protein arrays were scanned on a confocal microarray reader (ScanArray 4000, Perkin Elmer Life Science) and analyzed using the GenePix Pro 6.0 microarray image analysis software (Molecular Devices).

The Luminex-bead technique for autoantigen profiling was established because it has several advantages compared to the initially used protein array technique such as higher dynamic range, lower coefficients of variation, increased stability due to covalently linking of proteins and better performance in high-throughput analysis. For the bead autoantigen array 3061 autoantigens were selected. His-tag affinity purified recombinant proteins were covalently coupled to magnetic carboxylated color coded MagPlex^TM^ microsphere beads following the manufacturer’s protocol (Luminex). For each single coupling reaction up to 12.5 μg antigen and 8.8 x 10^5^ individually coded beads were used. The coupling efficiency and bead stability were monitored. One bead subarray consisted of up to 384 different antigen-coated beads and eight control beads. Due to the total number of antigens, eight different subarrays were established and used for the analyses. Protein-coated beads were distributed into 96-well microtiter plates and incubated with diluted (1:100) serum samples for 22 hrs at 4°C. On each assay plate, three reference sera were measured serving as quality control. Unbound antibodies were removed by washing. Bound human antibodies were quantified by probing with phytoerythrin (PE)-labeled anti-human detection antibody (goat-anti-human-PE, Jackson/Dianova) followed by several washing cycles and measurement of the fluorescent signal on a FlexMap3D device (Luminex) (DD gate 7.500–15.000; sample size: 80 μl; 1000 events per bead region; timeout 60 sec).

### Data pre-processing and biostatistical analysis

#### Planar protein arrays

After median background subtraction, the median fluorescence intensity of 4 replicate spots was calculated for each autoantigen and normalized to spotted IgG controls. Cut-offs were determined individually for each autoantigen and calculated as the mean signal intensity of the low inflammation control cohort plus 3 standard deviations. High inflammation cohort samples with values above the cut-off were classified as positive and all antigens were sorted according to descending numbers of positive samples. N-fold increase or decrease of a specific autoantibody was determined on the basis of the normalized mean signal intensity in the high compared to the low inflammation cohort. Ranking of top candidate markers was based on fold-change and corrected p-Value.

#### Bead protein arrays

The measured median bead fluorescence intensity for each autoantigen was used for further calculations. In rare cases when less than 10 beads were retrieved for measurement, this value was set to missing. These values were replaced by the median value of this autoantigen measured in all samples. Autoantigens with more than 20% missing values were excluded from further analysis. After log2 transformation quantile normalization [43, 44] was used to normalize samples on each individual plate.

The classification of the five best performing autoantibodies identified with the bead array technique for differentiation of the low and the high inflammation cohorts was based on a logistic regression model with the group variable (high/low inflammation) as the dependent variable and the mean fluorescence values of the autoantibodies as the influencing factors. P-values for single influencing factors were determined based on the Wald test, and additionally parameter estimates and the respective 95% confidence intervals (CI) two-sided were calculated. Odds ratios (OR) were given together with the respective 95% confidence intervals two-sided. The classification performance was assessed based on the sensitivity, specificity, and AUC values that were achieved with this model [43, 45]

### Functional annotation and pathway analysis

After mapping the top 165 autoantibodies tested positive with the protein microarray to genes, a set of 136 genes was obtained and used for gene ontology analysis. Functional annotation clustering was performed using the DAVID database (http://david.abcc.ncifcrf.gov) [46, 47].

### Tissue Microarray (TMA) and immunohistochemistry

For the construction of a tissue microarray (Inflammation-TMA) formalin fixed and paraffin embedded human tissue samples (n = 70) displaying high or low amounts of tissue infiltrating lymphocytes were selected from the autoantibody screening cohort. Clinical and pathological characteristics are summarized in **Table 1**in column “Screening Cohort”. The use of archived samples deriving from radical prostatectomy specimens obtained at the University Hospital Innsbruck was approved by the Ethics Committee of Medical University of Innsbruck. For each case three cancer tissue cores and three benign cores with a diameter of 0.6 mm were punched out of the donor tissue and transferred to the recipient TMA block [48]. The TMA was assembled using a manual tissue arrayer (Beecher Instruments, Sun Prairie, WI). Basal cell marker p63 and tumor cell marker α-methylacyl-CoA racemase (AMACR) stainings used to control the histological diagnosis and SPAST, STX18 or SPOP stainings, respectively, were performed on a Discovery-XT staining device (Ventana, Tucson, AZ) using instrument standard protocols. Target antibodies, suppliers, article numbers, and concentrations used were as follows: anti-SPAST, Atlas Antibodies (Stockholm, Sweden), #HPA017311, 1:50; anti-STX18, Atlas-Antibodies, #HPA003019, 1:150; anti-SPOP, Sigma-Aldrich (St. Louis, MO), #SAB1406659, 1:50; anti-p63, Sigma-Aldrich, #P3362, 1:200; anti-AMACR, Dako (Vienna, Austria), #M3616, 1:200, anti-CD45, Dako, #M0701, 1:300.

**Table 1:** Clinical and pathological cohort characteristics.

Parameter	Screening Cohort		Cross-Validation Cohort	
AAB detection technique	Protein Array		Luminex	
Inflammation state	high inflam	low inflam	high inflam	low inflam
Number of patients	38	32	33	30
Age [yrs]	62 ± 6.5	58 ± 8.4	60 ± 6.4	56 ± 6.3
C-reactive protein [mg/l]	0.27 ± 0.31	0.26 ± 0.34	0.37 ± 0.64	0.48 ± 0.27
Tumor grade: Gleason score	7 ± 0.9	7 ± 0.8	7 ± 1.0	6 ± 0.7
GSC 5 [%]	2.6	3.1	3.0	6.7
GSC 6 [%]	18.4	25.0	48.5	46.6
GSC 7 [%]	63.2	65.6	33.3	40.0
GSC 8 [%]	2.6	0.0	6.1	6.7
GSC 9 [%]	13.2	6.3	9.1	0.0
Prostate volume [ml]	46 ± 16.5	39 ± 9.3	34.2 ± 10.6	34.4 ± 8.8
PSA level [ng/ml]	5.2 ± 3.0	6.4 ± 8.1	4.1 ± 1.7	3.8 ± 1.5
0.0–2.0 ng/ml [%]	5.3	18.7	0.0	0.0
2.0–4.0 ng/ml [%]	31.6	34.4	66.7	73.4
4.0–10.0 ng/ml [%]	57.8	37.5	33.3	26.6
>10.0 ng/ml [%]	5.3	9.4	0.0	0.0
fPSA [%]	18 ± 7.5	16 ± 7.2	17 ± 7.0	14 ± 4.8

Mean values and standard deviations or % distributions, respectively, are displayed. PSA, prostate specific antigen; fPSA%, % of free PSA compared to total PSA (free + protein bound); GSC, Gleason score, the sum of the two most prevalent Gleason patterns, higher Gleason score characterizes higher dedifferentiation and aggressiveness of tumor cells.

Evaluation of immunohistochemical staining intensities was supervised by an experienced uropathologist (G.S.). Images were acquired using an Axio Imager Z2 microscope (Zeiss) and TissueFAXS software (TissueGnostics). Quantitative immunohistochemical analysis was performed using the HistoQuest immunohistochemistry analysis software (TissueGnostics). For each TMA spot the mean intensity and percentage of positively stained cells were evaluated and a score was calculated by multiplying those two values for each TMA core. Mean score values were calculated from three cancer and three benign tissue cores of each patient. Mann Whitney U test was used for the analysis of differences between the two groups.

A second TMA (Targos-TMA) comprising 111 tissue specimens from histological normal prostate (BE), benign prostate hyperplasia (BPH), prostate carcinoma (CA) as well as clinically diagnosed castration refractory tumors (CRPC), was constructed and stained as described above. The use of these tissue samples deriving from radical prostatectomy surgeries at the Hospital of Kassel was approved by the institutional review boards. Stainings were evaluated by an independent pathologist (M.K.), using a semiquantitative scoring system (H-Score), which combines four intensity categories with the estimated percentage of stained cells [49]. Mann Whitney U test was used for the analysis of differences between groups.

### RNA isolation and qPCR

Total RNA was extracted from benign and malignant areas of frozen tissue sections of both patient groups (high/low inflammation, n = 66) using the AllPrep DNA/RNA Micro Kit (Qiagen, GmbH, Hilden, Germany). RNA concentrations and purity were determined spectrophotometrically. Reverse Transcription (RT) was performed on 500ng of total RNA using iScript select cDNA synthesis kit (Bio-Rad, Hercules CA, USA) and random hexamer primers (Promega, Madison WI, USA). QPCR (40 cycles) was performed in triplicates using 8ng total RNA equivalents of cDNA for each 10μl reaction. The following Taqman assays (Applied Biosystems, Foster CityCA, USA) were used: PTPRC, Hs04189704_m1; STX18, Hs01099207_m1; SPOP, Hs00737433_m1; SPAST, Hs00208952_m1; TBP, Hs00427620_m1. Target gene expression was normalized to the housekeeping gene TBP. The relative expression ratio (R) was computed based on Taqman assay efficiency of target gene and reference gene and the cycle threshold (Ct) deviation of each sample to a control sample (calibrator, cDNA mix of 3 benign and 3 malignant tissue sections, 20ng qPCR input) using the following formula: R = [E_target_ ^ΔCt (calibrator–sample)] / [E_reference_ ^ΔCt (calibrator–sample)][50].

### Search for SPOP mutations

Fifty-three cDNA samples deriving from RNA isolation of high/low inflammation tissue specimens described in the previous paragraph were used for the SPOP mutation screen. A primer pair (Eurofins MWG Operon, Ebersberg, Germany) flanking the region of recurrent SPOP mutations was designed using the open-source software Primer 3: Fwd, 5’-AAGGGTTCCAAGTCCTCCAC-3’; Rev, 5’-CGGCACTCAGGAACCTTTAC-3’ [51]. PCR amplification was performed on 100ng of total RNA equivalent with Taq DNA Polymerase (Peqlab, Erlangen, D) in a total of 100μl, applying the following conditions: denaturation at 95°C for 2 min; 40 cycles of 95°C for 1 min, 62°C for 30 sec, and 72°C for 1 min; elongation at 72°C for 7 min. PCR product quantity and size was checked on 1.2% Agarose Flash Gels (Lonza, Rockland, USA). DNA was purified using the Qiagen PCR extraction kit and sequenced using the same primers according to a standard Sanger sequencing protocol (Mycrosynth, Balgach, CH).

Since the frequency of detected SPOP mutations was significantly lower than the one reported in the literature [51], results were confirmed by the method originally described by Blattner et al. [52], DNA was isolated from benign and malignant areas of frozen tissue sections using the AllPrep DNA/RNA Micro Kit. After an initial pre-PCR amplification step to enrich the target regions, a high-resolution melting analysis (HRM) was carried out. This assay targets exons 6 and 7 of the SPOP gene containing all previously detected mutations in prostate cancer (amino acids 80 to 106 and amino acids 120 to 140). The sample that showed a notable shift in the melting curve was sent off for Sanger sequencing to confirm and further specify its alteration.

## Results

### Serum autoantibody levels are elevated in prostate cancer patients with immune cell infiltration of the prostate

In an attempt to identify prostate chronic inflammation associated autoantibodies, prostate cancer patients displaying a low or a high number of infiltrating immune cells in their radical prostatectomy specimens were subjected to analysis. In order to identify high and low inflammation patient cohorts, we took advantage of the number of infiltrating immune cells throughout the whole prostate. For each patient twenty to thirty tissue slides representing different areas of the prostate were stained for the pan leukocyte marker CD45 to define tumor-infiltrating lymphocytes. An experienced uropathologist conducted the classification into two groups (high/low inflammation) according to a histopathological classification system for chronic prostatic inflammation [53]. Patient samples with no or mild inflammation were specified as “low inflammation group” and those with moderate and high inflammation as “high inflammation group”. Clinical parameters including age, inflammation marker C-reactive protein(CRP), Gleason Score, prostate volume, PSA and free PSA were equally distributed amongst the high and the low inflammation groups (**Table 1, S1 Fig**).

**Fig 1:**
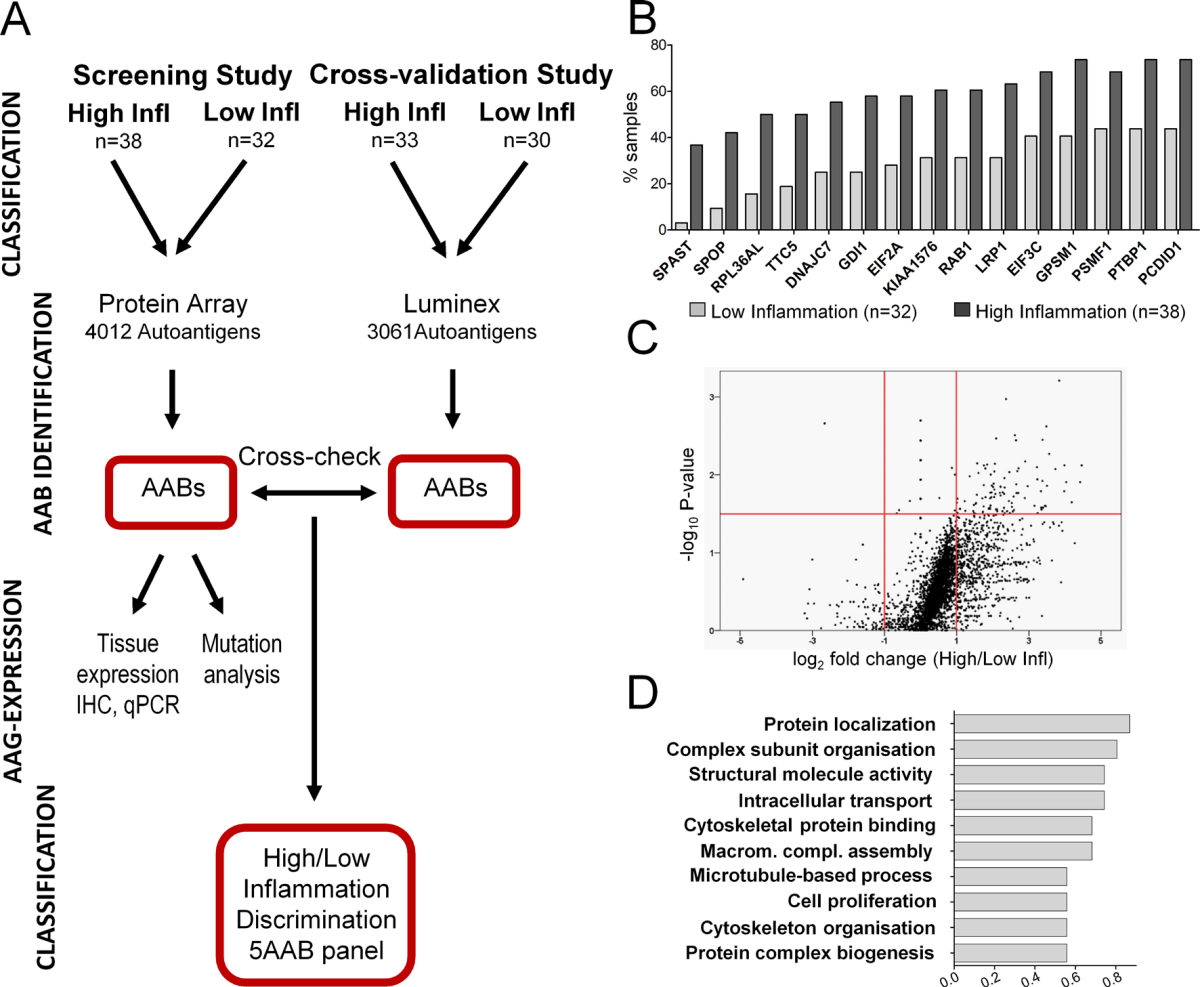
Chronic prostatic inflammation induces elevated autoantibody levels. **A** Flow chart of the strategy used for the detection and cross-validation of autoantibody (AAB) signatures associated with chronic prostatic inflammation. Radical prostatectomy specimens were classified into two (high/low inflammation) groups based on the extent of immune cell infiltrations in the whole prostate. The corresponding pre-surgery blood serum samples were analyzed for autoantibodies (AAB) using a planar protein array (screening, n = 70). A cross-validation study testing the robustness of the identified AAB panel was based on the Luminex-bead protein array technology (cross-validation, n = 63). Statistical comparison of the serum autoantibody profiles in the low and high inflammation groups was used to identify and validate differentially abundant AABs. The prostate tissue expression patterns and the expression in different prostate cancer progression stages were established for three selected corresponding autoantigens (AAGs). **B** Bar chart for positively classified observations of the 15 most differentially detected autoantibodies in the high inflammation group compared to the low inflammation group. Data are expressed as percentage of total number of positive samples in each group. **C** Calculation of the fold change for each autoantibody revealed a significant increase of 165 antigens in high inflammation prostate cancer (upper right panel, p<0.05, fold change>2, Mann-Whitney Test) and a decrease of only one (upper left panel). **D** Graphical representation of the ten top ranked functional clusters assigned for inflammation associated autoantibodies using the DAVID functional annotation tool. The bar size corresponds to the percentage of identified corresponding genes related to a specific functional category (P<0.05).

The serum autoantibody profiles of 38 high inflammation and 32 low inflammation (control) patients were obtained for corresponding pre-surgery blood serum samples employing a 4012 recombinant protein array screen **(Fig 1A)**. Selection of the antigen test panel was based on own and reported data on autoantigens associated with prostate cancer, other types of cancer and inflammation-related diseases such as multiple sclerosis or lupus erythematosus, respectively [31, 36–42] **(S1 Table).** Autoantibodies to 3919 autoantigens were detected in at least one of the patients and the number of patients tested positive for autoantibodies corresponding to an individual autoantigen ranged from 0 to 69 of 70 **(S1 Table)**. Considering the number of positive samples in both groups generated a list of 15 autoantibodies most differently present in the high compared to the low inflammation group (**Fig 1B**). The top-ranked autoantibodies against spastin (SPAST, giI40806168) and speckle-type POZ protein (SPOP, giI56117827) were present in 37% and 42% of sera deriving from high inflammation patients whereas detectable in less than 3% and 10% of the sera from low inflammation control patients. Evaluation of the fold-change for each of the 997 autoantibodies in the high compared to the low control inflammation group, revealed significantly increased abundance (p<0.05) of 165 autoantibodies in the high inflammation patients’ cohort (**Fig 1C, S2 Table**). None of them was determined exclusively within the high inflammation group. Interestingly, the intensity level of only one autoantibody (binding antigen FEZF2,giI157388917) was significantly decreased (log2-foldchange: -2.66, p-Value: 0.002) in the high compared to the low inflammation control group.

We wondered whether the 165 autoantibodies significantly enhanced by chronic inflammation are associated with distinct functional clusters and therefore mapped them to their corresponding genes to perform a functional annotation clustering using the DAVID database. Several enriched molecular functions and biological processes were identified. Of the top ten annotation terms “protein localization” formed the biggest cluster containing 14 associated genes coding for proteins involved in vesicular trafficking (GDI1, MYH9), endo- (CDC42) and exocytosis (STX18), chromatin binding (CHMP5) and cytotoxic T-cell activation (CTLA).The cluster “macromolecular complex subunit organization” was composed of genes required for DNA repair (SF3B3), protein synthesis (EIF2A), T-cell proliferation (FADD), and microtubule disassembly (STMN1, SPAST). Overall, we observed that autoantibodies overrepresented in high inflammation patients were mainly directed against structural antigens and cell proliferation associated proteins (**Fig 1D, Table 2**).

**Table 2:** Functional annotation of the top biomarker candidates.

Functional cluster	GO_ID	Count	%	p-Value	Genes
Protein localization	GO:0008104	14	0.867	0.0139	GDI1, CLTA, NFKBIE, CHMP5, NAPA, MYH9, RAB11FIP4, CDC42, STX18, KIFAP3, CD81, RAB11B, GNAS, GOSR2
Macromolecular complex subunit organization	GO:0043933	13	0.805	0.0065	WASF1, FADD, EVL, EIF2A, CDK7, SF3B3, SDHAF1, MAZ, KIFAP3, SNRNP200, STMN1, TUBA1A, SPAST
Structural molecule activity	GO:0005198	12	0.743	0.0060	RPL35A, NUMA1, CLTA, CLDN9, RPS16, MYL6B, MRPS24, LMNA, RPL27, TUBA1A, RPL21P16, RPL36AL
Intracellular transport	GO:0046907	12	0.743	0.0098	CLTA, KIF5B, STX18, MYL6B, CHMP5, SLC25A6, GOSR2, GNAS, NAPA, MYH9, FTH1, SPAST
Cytoskeletal protein binding	GO:0008092	11	0.682	0.0035	NUMA1, TWF2, KIF5B, WASF1, KIFAP3, EVL, STMN1, MYH9, COTL1, SPAST, FARP2
Macromolecular complex assembly	GO:0065003	11	0.682	0.0266	MAZ, WASF1, KIFAP3, SNRNP200, EIF2A, FADD, CDK7, TUBA1A, SDHAF1, SF3B3, SPAST
Microtubule-based process	GO:0007017	9	0.558	0.0006	KIF5B, KIFAP3, KIF18B, STMN1, MYH9, DYNC1H1, TUBA1A, SPAST, DCTN2
Cell proliferation	GO:0008283	9	0.558	0.0162	PRPF19, LRP1, CD81, ZNF259, CDK7, CSRP2, FTH1, LRPAP1, DCTN2
Cytoskelet onorganization	GO:0007010	9	0.558	0.0162	CDC42, NISCH, WASF1, EVL, STMN1, MYH9, DYNC1H1, SPAST, DCTN2
Protein complex assembly	GO:0070271	9	0.558	0.0350	MAZ, WASF1, KIFAP3, FADD, CDK7, TUBA1A, SDHAF1, SF3B3, SPAST

%,.number of genes associated with functional cluster/total number of query genes

### Antigens of serum-derived autoantibodies are expressed in the prostate

To elucidate whether antigens of identified autoantibodies are expressed and possibly dysregulated in prostate tissue, we investigated the expression pattern of distinct target proteins. For that three candidate antigens, Spastin (SPAST), speckle-type POZ protein (SPOP), and syntaxin 18 (STX18), were selected according to the following criteria: (i) autoantibodies are amongst the top differentially abundant inflammation–associated ones according to p-values and fold change (**S2 Table**); (ii) antibodies for IHC are commercially available and the corresponding antigen levels are sufficient for immunohistological detection according to the Human Protein Atlas (www.proteinatlas.org); (iii) associations with different cancer types had been reported (**Table 3**). A tissue microarray including tissue samples of high and low inflammation patients of the autoantibody screen was generated and used for immunohistochemical detection of these autoantigen proteins.

**Table 3:** Candidate autoantigens selected for analysis of their tissue expression pattern.

Autoantigen	GI Accession	p-Value	Fold change	Association with cancer
SPAST	gi|40806168	0.001	14.33	[54–56]
SPOP	gi|56117827	0.003	4.27	[57–59]
STX18	gi|39725935	0.014	7.82	[60]

**Fig 2:**
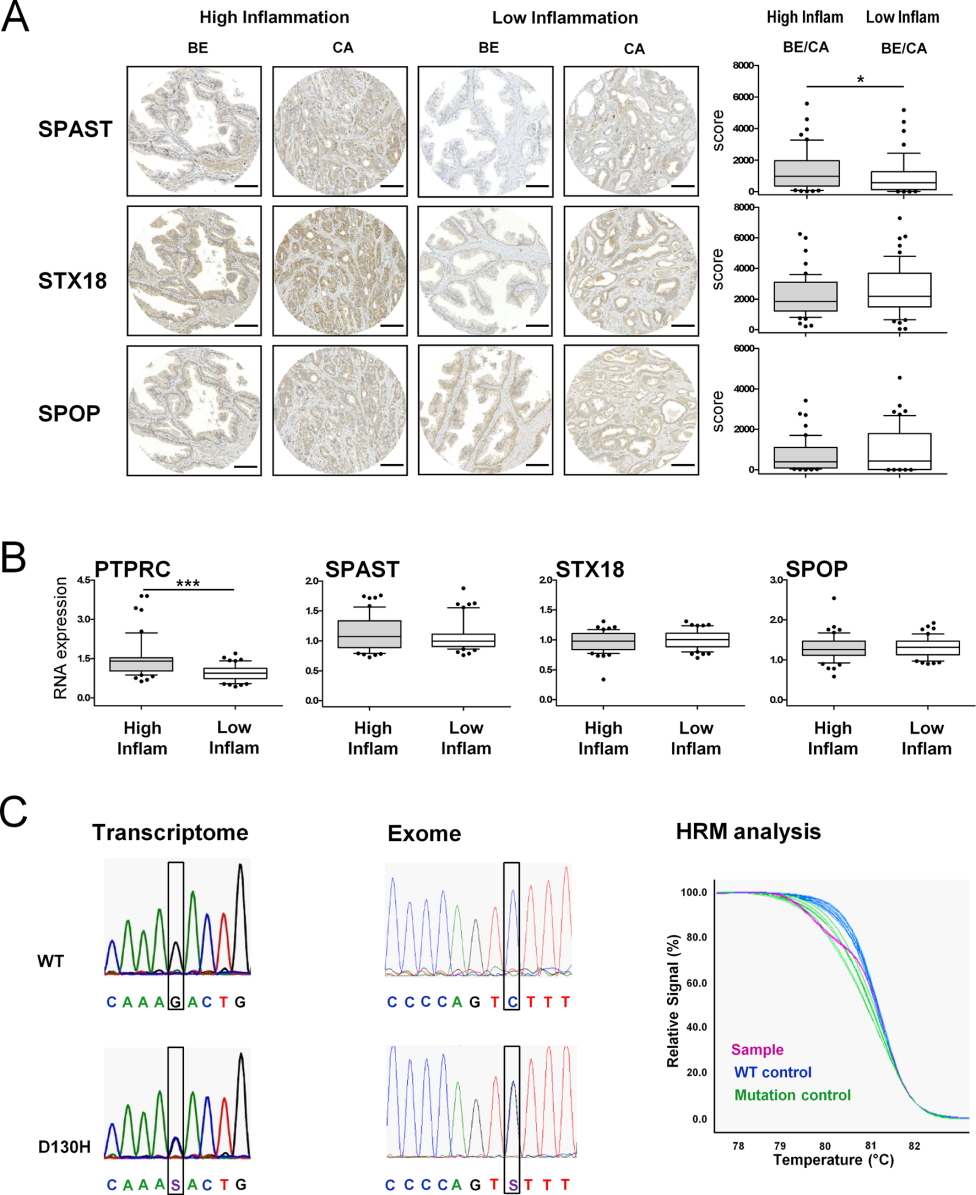
Quantification of corresponding autoantigen levels in prostate tissue. **A** Immunohistochemical stainings of representative tissue microarray spots from high and low inflammation patient cohorts. SPAST, STX18 and SPOP are expressed in the epithelium of benign (BE) and cancer (CA) areas of both cohorts. Quantitative analysis was performed using the HistoQuest immunohistochemistry analysis software (TissueGnostics). A score was calculated by multiplying staining intensity and percentage of positively stained cells. n = 25 per group. *P<0.05, Mann-Whitney Test. Bar, 100μm. **B** Quantification of the pan-lymphocyte marker CD45 (PTPRC) and autoantigen mRNA levels in high and low inflammation patient groups. n = 25 per group. ***P<0.001, Mann-Whitney Test. **C** Electropherograms of transcriptome (5’-3’) and exome (3’-5’) sequencing results depicting wild-type and D130H SPOP mutation sequences. High resolution melting curves for mutated and wild-type DNA: The purple melting curve of the sample consists of 50% mutant and 50% wild-type DNA.

SPAST, SPOP and STX18 were found expressed in the epithelium of benign and cancer areas in both patient cohorts. Quantification of immunostaining intensities revealed a significantly increased SPAST expression in the high inflammation compared to low inflammation prostate tissue samples. However, SPOP and STX18 immunoreactivities were unaltered between high and low inflammation patient samples (**Fig 2A**).

As expected, mRNA levels of the common inflammation marker PTPRC (CD45 antigen coding gene) were significantly increased in high inflammation tissue sections (**Fig 2B**). Quantification of SPAST, STX18 and SPOP mRNA by qRT-PCR identified similar expression levels between high and low inflammation tissue sections, confirming immunohistochemistry data with the exception of SPAST. Consequently, the validation of autoantibody targets in prostate tissue revealed the expression of antigens of circulating autoantibodies, however, protein and mRNA expression in the prostate were not explicitly related to serum autoantibody levels.

### Are autoantibodies triggered by protein mutations in prostate tumors?

Given our finding that autoantigen abundance and expression pattern in prostate tissue was found only moderately different in high versus low inflammation PCa cases, the question arises, what triggers generation or higher abundance of autoantibodies. Mutant proteins might be one possible cause for the amplification of an autoimmune response. For example, mutant forms of the p53 protein elicit anti-p53 antibodies in 30 to 40 percent of patients with various types of cancers [40]. In line with this observation one of our top autoantigen candidates, SPOP, was identified as one of the few proteins recurrently mutated in prostate cancer. Up to 13% of prostate tumors were reported to harbor SPOP mutations and this tumor subtype shows a distinct pattern of genomic alterations [51]. We hypothesized that abundant SPOP autoantibodies might be triggered by mutant protein variants in the corresponding tumors. To investigate this hypothesis we searched for SPOP mutations in our high and low inflammation tumor cohorts. RNA samples of 51 high and low inflammation tumor tissues of the patient cohort profiled for autoantibodies were screened via Sanger sequencing of a PCR-amplified SPOP c-DNA fragment. A D130H mutation was identified in one of the prostate cancer tissue samples (**Fig 2C**). The observed mutation frequency was lower than expected according to literature data. Therefore we repeated the analysis using the previously published method of high resolution melting analysis of genomic DNA fragments and subsequent Sanger sequencing of identified samples [52], and obtained identical results (**Fig 2C**). Measured SPOP autoantibody levels were very low in the corresponding serum sample of this tumor case and we thus were not able to confirm a link between mutant SPOP and occurrence of circulating SPOP autoantibodies.

### Autoantigen expression pattern in different stages of prostate cancer

To test whether selected autoantigens are differentially expressed in different progression stages of prostate cancer, we undertook a comparative immunohistochemistry analysis of SPAST, SPOP and STX18 employing an independent TMA comprising 111 tissue specimens from histological normal prostate (BE), benign prostate hyperplasia (BPH), prostate primary carcinoma (CA) as well as from clinically diagnosed castration resistant prostate cancer (CRPC). Analysis of protein expression within matched pairs of benign prostate tissue and corresponding tumor areas revealed an increased expression of SPAST and STX18 in most tumor samples whereas SPOP expression remained unchanged. Interestingly, tumors derived from endocrine therapy resistant patients (CRPC) were negative for SPAST and SPOP, suggesting loss of these two autoantigens during tumor progression (**S2 Fig**).

### Cross-validation of the high inflammation prostate cancer signature

To test the robustness of the identified autoantibody marker set, an independent cross-validation study was performed. For that we used an autoantibody profiling platform based on antigen-coated, color-coded Luminex™ magnetic beads. This method was developed as it has several advantages compared to the initially used planar protein microarray, which showed limitations concerning small production batches, batch differences, low automation grade and consequently low sample throughput. In contrast, bead-based protein arrays allow high sample throughput, large batch sizes and a high level of automatization. Moreover, this platform is better suitable to transfer an assay into a clinical setting. The bead protein array was established as a broadly applicable autoantibody analysis tool and consisted of 3065 individual protein beads grouped into 8 subarrays. As for the planar protein array, selection of autoantigen proteins was based on published data [31, 33–42] considering also autoantigen protein production and performance. The final autoantigen panel comprised putative biomarkers for autoimmune diseases and tumor-related processes (**S1 Table**). Based on the corresponding Gene ID´s, 84% of the antigens used for the planar protein microarray were also represented in the protein bead array. Of the top 165 differentially abundant antigens detected by the planar protein microarray technique 129 were included in this novel autoantigen panel, however, one of our studied autoantigens, SPAST was missing.

**Fig 3:**
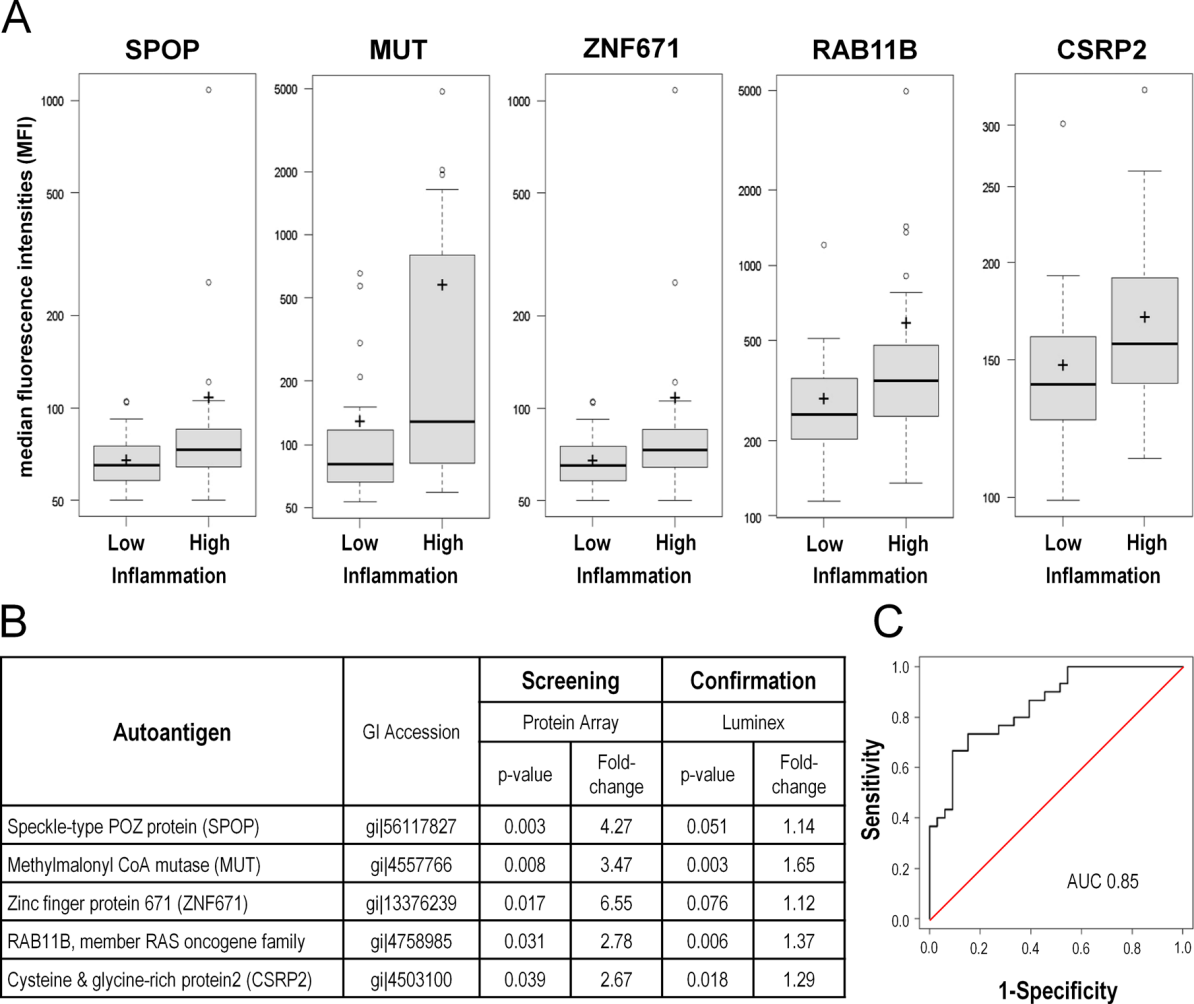
Cross-validation of identified autoantibody profile using a Luminex-beads protein array. The identified profile was validated in an independent set of prostate cancer patients (n = 60) using the bead-based Luminex technology to identify autoantibodies. **A** Box plots of mean fluorescent intensities (MFI) values for the five top autoantibody candidates significantly increased in the prostate high inflammation group in both screens. **B** Table displaying fold-change and p-values of the autoantibodies significantly upregulated in high-inflammation serum samples of the screening and validation patient cohorts. **C** ROC curve for the top five autoantibodies for the classification of samples of the validation set. The identified biomarker profile discriminates between high and low inflammation patients with an AUC of 0.85.

A new set of 33 serum samples from prostate cancer patients with inflammatory tissue infiltrates and 30 serum samples from patients with low inflammation were profiled with the bead array technique. Mean autoantigen fluorescence signals were calculated for each autoantigen for both groups Based on discriminatory performance criteria such as p-value, fold-change, AUC, and Cohen´s d, 51 antigens of the top 165 differentially abundant antigens detected in the initial protein array screen were retrieved in the cross-validation study. Autoantibodies against five proteins were considerably upregulated in both autoantibody profiles in the high inflammation group: SPOP, MUT, ZNF671, RAB11B and CSRP2 (**Fig 3A and 3B**).

Subsequently, we evaluated whether these five best performing antigens might be useful to discriminate between inflammatory states of the prostate. Classification performance was assessed based on a logistic regression model. This analysis was purely exploratory taking the 63 patient samples of the validation study into account. The ability to distinguish between prostate cancer patients with low inflammation from those with high inflammation attained a diagnostic specificity of 67% and sensitivity of 80%. The calculated receiver operating characteristic (ROC) curve reached an area under the curve (AUC) of 0.85 (**Fig 3C**).

These results demonstrate a certain stability of inflammation related autoantibodies amongst different patient cohorts and distinct detection methods. The identified biomarker panel consisting of five serum-derived autoantibodies has the potential to discriminate between high or low inflammation in prostate cancer patient samples.

## Discussion

To date several theories describe the cellular and molecular processes underlying the pathogenesis of prostate cancer. Multiple lines of evidence indicate that inflammation, which is very common within the adult prostate, represents an important factor in influencing prostatic growth [61, 62]. In addition, accumulating findings indicate that an inflammatory microenvironment supports the development of malignancy and progression to metastatic disease [17–19]. The detection and therapy of chronic prostatitis therefore represents a crucial step in the treatment of benign and early malignant prostate disease. However, in the absence of a validated biomarker, the histological examination of prostate biopsies remains the only way for identification of prostate inflammation [63]. A less invasive method would be of great help in patients’ care.

Observations that cancer is immunogenic with patients eliciting and potentially amplifying an immune response against their tumor antigens suggests that next to cytotoxic T-cells autoantibodies play a significant role in this setting [42, 63–65]. Typically, inflammatory infiltrates in the prostate are composed of T-lymphocytes (70%), B-lymphocytes (15%) and macrophages (15%) [63].

We hypothesized that patients with chronic inflammation might have a specific immune response to inflammation-related and prostate-associated proteins. We therefore screened prostate cancer patients grouped into low or high numbers of prostate infiltrating immune cell cases for the presence of circulating autoantibodies directed against a previously identified prostate cancer, other cancer type and autoimmune disease-associated autoantigen panel [31, 33–42]. Focusing on a cohort of cancer patients who underwent radical prostatectomy allowed to inspect the whole gland for infiltrating immune cells and guaranteed an accurate discrimination of high and low prostate inflammation patients. Identification of 165 antibodies significantly increased in high inflammation samples implied that a subset of serum autoantibodies is considerably amplified upon prostate inflammation, most probably due to the involvement of their target proteins in the inflammatory process. The 15 most differently positive autoantibodies were detected in about 40–70% of high and in 3–40% of low inflammation samples. The frequencies in the low inflammation group exhibited a similar range as previously reported for autoantibodies best performing in the discrimination of prostate cancer and benign patients [31]. Inflammation seems to further increase the likelihood of autoantibody positivity, whereas a lower frequency was reported for benign or healthy men [31].

Functional annotation analysis revealed that the corresponding autoantigens were mainly directed against cell structure and proliferation associated proteins. Cellular processes like “protein localization”, “intracellular transport”, “cytoskeleton organization” and “cell proliferation” were highly enriched in patients with inflammation. This observation further supports earlier findings that cytokines induced by inflammatory processes trigger local growth factor production, support angiogenesis and stromal cell proliferation in prostate tissue [66–69].

None of the identified autoantibodies in the current report was exclusively detected within the high inflammation group, however, several were significantly increased in the circulation of high inflammation prostate cancer patients. These findings are in line with former suggestions that circulating autoantibodies indicate an immune response to prostate tissue antigens [70]. In order to test this hypothesis, we investigated the expression of three selected target candidate autoantigens in prostate tissue. Immunohistochemistry and qPCR revealed that SPAST, STX18 and SPOP were expressed in the epithelium of benign and cancer areas of the high and the low inflammation cohorts. Only one of the three autoantigens, SPAST, was more abundant in high compared to low inflammation tissue samples suggesting that not primarily autoantigen expression levels but additional factors are crucial for stimulating the immune system to produce autoantiboides, e.g. cytokines produced in the inflamed microenvironment.

Protein expression levels of all three analyzed autoantigens, SPAST, STX18 and SPOP were significantly deregulated either in primary prostate tumors and/or in late, castration-resistant tumor stages. Thus proteins associated with malignancy and tumor progression seem to be potent autoantigens, in line with the hypothesis of an association of inflammation with the development of solid tumors including prostate cancer [71–73].

A number of further studies addressed the importance of mutations and gene variants on inflammation and prostate cancer risk [74, 75]. Despite gene rearrangements, such as the TMPRSS2-ERG genes fusion [76], recurrent mutations in the speckle-type POZ (SPOP) gene occur in up to 15% of prostate cancers making SPOP the most commonly affected gene by nonsynonymous point mutations in prostate cancer [52]. Recent findings suggest a critical tumor suppressor role of wild-type SPOP that is abrogated by prostate-cancer associated mutations of the gene [77]. As SPOP was one of our top autoantibodies in prostatic inflammation, we investigated a possible association of increased autoantibody levels with SPOP mutations and identified a D130H missense mutation in one patient sample. This mutation affects a conserved residue in the structurally defined substrate-binding cleft suggesting consequences on the tumorigenic phenotype. However, the low autoantibody level in the corresponding serum sample and the rare frequency of SPOP mutations in the study cohort indicate no crucial importance of mutations on serum-autoantibody levels in prostate cancer.

Variation of autoantibody signatures across different studies, patient cohorts and detection techniques is a major obstacle for development of diagnostic assays based on these markers. To test the robustness of the autoantibody signature established in the protein array screen we cross-validated the identified profile in a second independent patient cohort applying an improved technology that exhibits several advantages over planar arrays such as sensitivity, dynamic range and flexibility [78]—a Luminex-bead based autoantigen protein array, which became available after the initial protein array autoantibody profiling study. We were able to retrieve 51 of 129 differentially abundant autoantigens identified in the high inflammation group in the initial test screen. This result indicates a certain robustness of inflammation-related autoantibodies across different patient cohorts and distinct detection methods.

The best performing discriminatory autoantibodies of both screens were directed against MUT, RAB11B, CSRP2, SPOP and ZNF671. The combination of these five top autoantibodies distinguished prostate cancer patients with low from those with high inflammation with a sensitivity of 80% and a diagnostic specificity of 67%. An area under the ROC curve of 0.85 provided evidence that the selected marker panel has discriminatory potential to identify patients with prostate inflammation. In view of the problems of identification of prostate immune infiltration by histopathological examination of biopsies, such as high invasiveness, sampling bias and high analytical efforts required, these autoantibody markers should be helpful. A combination with other markers such as PSA subtypes or other immune markers may further increase the discriminatory accuracy.

A similar approach for the identification of autoantibody biomarkers using serological identification of antigens by recombinant expression cloning (SEREX) identified prostatitis-specific IgGs against several proteins among them the protein NY-CO-7 (AF039689.1) [65]. Of notice, like SPOP, which we identified here, NY-CO-7 is an ubiquitin ligase [79]. It therefore seems likely that similar cellular processes associated with inflammation were identified in our analysis and that study. Successive studies will have to investigate the frequency of these autoantibodies using larger patient cohorts and including different malignancies in order to assess tissue specificity. As to the future aim of employing inflammation associated autoantibodies in diagnostic tests, the identified marker panel is a starting point but has to be further evaluated with regard to identifying inflammation in patients with benign prostatic disease and those with prostate cancer.

## Conclusions

The data presented provide evidence of an inflammation-specific autoantibody profile in prostate cancer patients and confirm the expression of the corresponding autoantigens in prostate tissue. Classification performance of the biomarker panel confirmed in a cross-validation study reached a diagnostic specificity of 67% and sensitivity of 80%. Yet, since this analysis was exploratory, further investigations have to be undertaken to elucidate the interconnections between chronic prostatic inflammation, the according autoantibody profile and its potential diagnostic applications. The inflammation autoantibody panel is a useful tool to study prostatic inflammation in clinical practice and assess its influence on the progression of prostate cancer and a possible protective effect of early medical treatment of chronic prostate inflammation. Identified autoantigens might also be considered as immunological targets for the development of immunotherapy regimens.

## Supporting Information

S1 Fig Clinical parameters are equally distributed between high and low inflammation patient cohorts. All samples were tested for statistically significant differences between the two sample groups. Clinical parameters including age, C-reactive protein, Gleason Score, prostate volume, PSA and free PSA (Table 1) are equally distributed amongst the high and low inflammation patient cohorts of the initial screen and the cross-validation set. P<0.05, Mann-Whitney Test.
(PDF)

S2 Fig Autoantibody marker antigens are differentially expressed in benign tissue and malignant lesions of the prostate. **A** Representative images for SPAST, STX18 and SPOP immunohistochemical double stainings with the basal cell marker p63. Left side shows benign lesions, right side represents invasive prostate carcinoma. The basal cell marker, which is absent in malignant glands, confirmed correct discrimination of benign and tumor regions. **B** Statistical distribution of candidate marker proteins in benign prostate (BE, n = 75), benign prostatic hyperplasia (BPH, n = 27), primary carcinoma (CA, n = 58) and castration resistant prostate cancer (CRPC, n = 9) samples. H-Scores were used for quantification of immunoreactivity. SPAST and STX18 and SPOP protein expression was significantly increased in tumors compared to benign or BPH tissue, respectively. In CRPC expression of SPAST and SPOP disappeared whereas expression of STX18 remained constant compared to the primary tumors.*P<0.05, **P<0.01, ***P<0.001, Mann-Whitney Test. Bar, 100μm.
(PDF)

S1 Table List of autoantigens included in the planar protein microarray (Table "Protein Array") and in the Luminex bead protein array (Table "Luminex"). Autoantigen proteins included in the planar protein array or in the Luminex bead protein array are listed. The number of positive samples of indivual auoantigens for the low and the high inflammation cohorts (colums D,E), p-values for the discrimination of the low and high inflammation samples (colums F) and mean fold changes, high compared to low inflammation samples (colums G) are listed. Autoantigens are ranked according to p-values.
(XLSX)

S2 Table List of 165 significantly upregulated autoantibodies in serum samples of the screening cohort, ranked according to p-Value. (XLSX)
