# Supplementary material for: Serum Autoantibodies in Chronic Prostate Inflammation in Prostate Cancer Patients
Source: PLoS One. 2016 Feb 10;11(2):e0147739. doi: 10.1371/journal.pone.0147739 (PMC4749310; doi:10.1371/journal.pone.0147739)
Supplement: S1 Fig — All samples were tested for statistically significant differences between the two sample groups. Clinical parameters including age, C-reactive protein, Gleason Score, prostate volume, PSA and free PSA (Table 1) are equally distributed amongst the high and low inflammation patient cohorts of the initial screen and the cross-validation set. P<0.05, Mann-Whitney Test. (PDF) [file pone.0147739.s001.pdf]

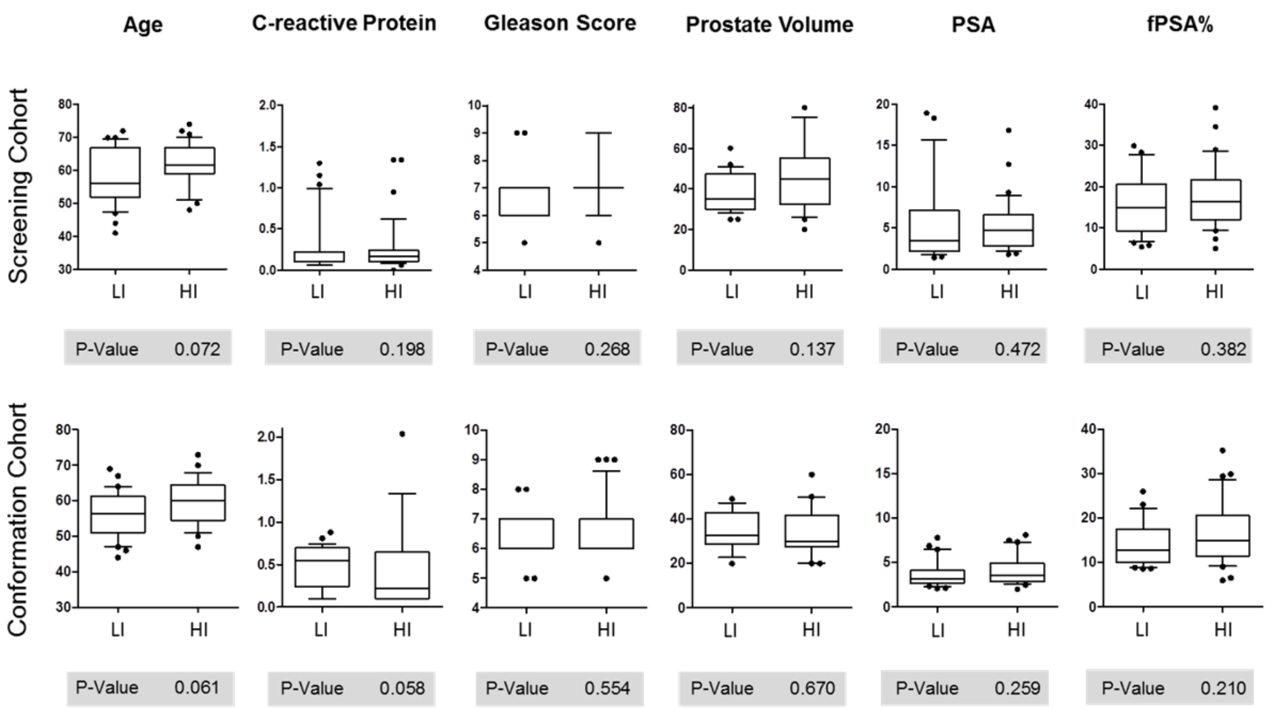

S1 Fig. Clinical parameters are equally distributed between high and low inflammation patient cohorts.

All samples were tested for statistically significant differences between the two sample groups. Clinical parameters including age, C-reactive protein, Gleason Score, prostate volume, PSA and free PSA (Table 1) are equally distributed amongst the high and low inflammation patient cohorts of the initial screen and the cross-validation set. $P<0.05$, Mann-Whitney Test.
