# Supplementary material for: Serum Autoantibodies in Chronic Prostate Inflammation in Prostate Cancer Patients
Source: PLoS One. 2016 Feb 10;11(2):e0147739. doi: 10.1371/journal.pone.0147739 (PMC4749310; doi:10.1371/journal.pone.0147739)
Supplement: S2 Fig — A Representative images for SPAST, STX18 and SPOP immunohistochemical double stainings with the basal cell marker p63. Left side shows benign lesions, right side represents invasive prostate carcinoma. The basal cell marker, which is absent in malignant glands, confirmed correct discrimination of benign and tumor regions. B Statistical distribution of candidate marker proteins in benign prostate (BE, n = 75), benign prostatic hyperplasia (BPH, n = 27), primary carcinoma (CA, n = 58) and castration resistant prostate cancer (CRPC, n = 9) samples. H-Scores were used for quantification of immunoreactivity. SPAST and STX18 and SPOP protein expression was significantly increased in tumors compared to benign or BPH tissue, respectively. In CRPC expression of SPAST and SPOP disappeared whereas expression of STX18 remained constant compared to the primary tumors.*P<0.05, **P<0.01, ***P<0.001, Mann-Whitney Test. Bar, 100μm. (PDF) [file pone.0147739.s002.pdf]

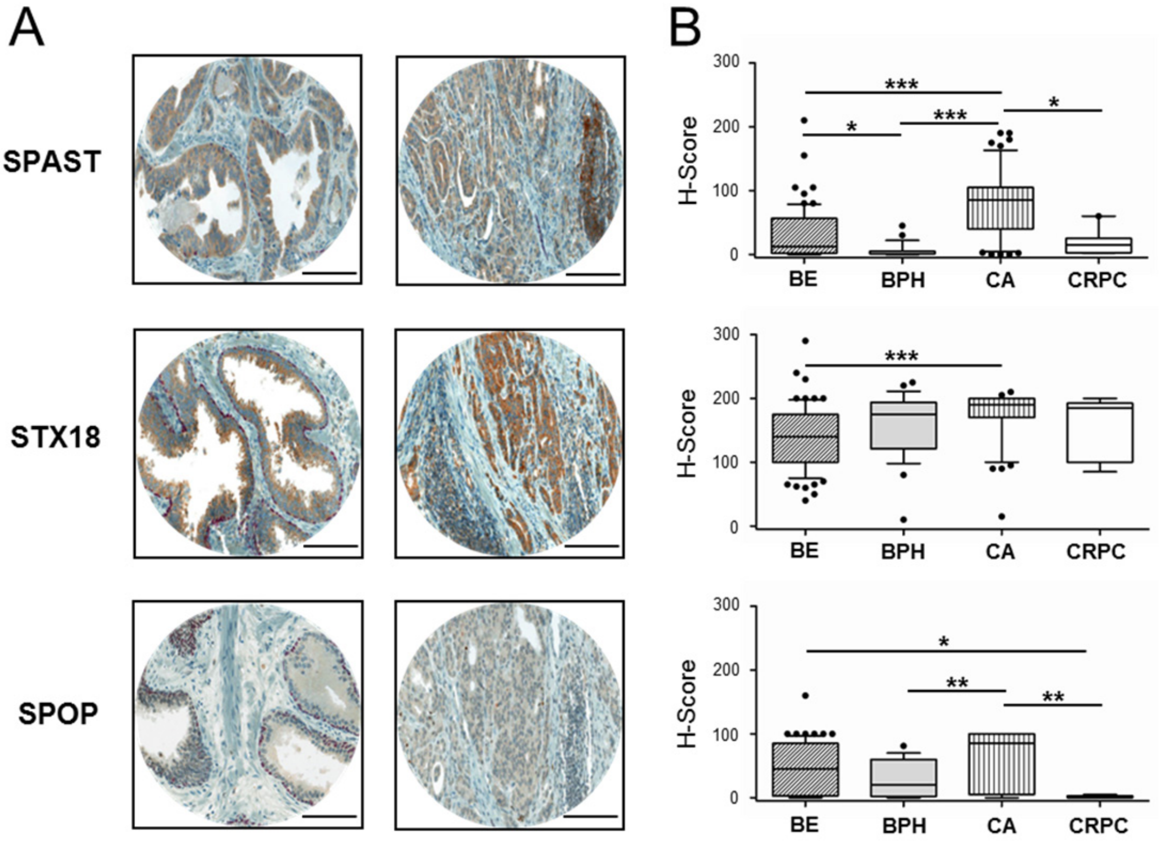

S2 Fig. Autoantibody marker antigens are differentially expressed in benign tissue and malignant lesions of the prostate.

A Representative images for SPAST, STX18 and SPOP immunohistochemical double stainings with the basal cell marker p63. Left side shows benign lesions, right side represents invasive prostate carcinoma. The basal cell marker, which is absent in malignant glands, confirmed correct discrimination of benign and tumor regions.
B Statistical distribution of candidate marker proteins in benign prostate (BE, n=75), benign prostatic hyperplasia (BPH, n=27), primary carcinoma (CA, n=58) and castration resistant prostate cancer (CRPC, n=9) samples. H-Scores were used for quantification of immuno-reactivity. SPAST and STX18 and SPOP protein expression was significantly increased in tumors compared to benign or BPH tissue, respectively. In CRPC expression of SPAST and SPOP disappeared whereas expression of STX18 remained constant compared to the primary tumors. *P<0.05, **P<0.01, ***P<0.001, Mann-Whitney Test. Bar, 100μm
